# Different human vaccine adjuvants promote distinct antigen-independent immunological signatures tailored to different pathogens

**DOI:** 10.1038/srep19570

**Published:** 2016-01-21

**Authors:** Niels Peter H. Knudsen, Anja Olsen, Cecilia Buonsanti, Frank Follmann, Yuan Zhang, Rhea N. Coler, Christopher B. Fox, Andreas Meinke, Ugo D´Oro, Daniele Casini, Alessandra Bonci, Rolf Billeskov, Ennio De Gregorio, Rino Rappuoli, Ali M. Harandi, Peter Andersen, Else Marie Agger

**Affiliations:** 1 Department of Infectious Disease Immunology, Statens Serum Institut, Copenhagen, Denmark; 2 Novartis Vaccines and Diagnostics s.r.l (a GSK Company), Siena, Italy; 3 Department of Microbiology and Immunology, University of Gothenburg, Gothenburg, Sweden; 4 Infectious Disease Research Institute, Seattle, WA, USA; 5 Valneva Austria GmbH, Vienna, Austria

## Abstract

The majority of vaccine candidates in clinical development are highly purified proteins and peptides relying on adjuvants to enhance and/or direct immune responses. Despite the acknowledged need for novel adjuvants, there are still very few adjuvants in licensed human vaccines. A vast number of adjuvants have been tested pre-clinically using different experimental conditions, rendering it impossible to directly compare their activity. We performed a head-to-head comparison of five different adjuvants Alum, MF59®, GLA-SE, IC31® and CAF01 in mice and combined these with antigens from *M. tuberculosis*, influenza, and chlamydia to test immune-profiles and efficacy in infection models using standardized protocols. Regardless of antigen, each adjuvant had a unique immunological signature suggesting that the adjuvants have potential for different disease targets. Alum increased antibody titers; MF59® induced strong antibody and IL-5 responses; GLA-SE induced antibodies and Th1; CAF01 showed a mixed Th1/Th17 profile and IC31® induced strong Th1 responses. MF59® and GLA-SE were strong inducers of influenza HI titers while CAF01, GLA-SE and IC31® enhanced protection to TB and chlamydia. Importantly, this is the first extensive attempt to categorize clinical-grade adjuvants based on their immune profiles and protective efficacy to inform a rational development of next generation vaccines for human use.

Beyond doubt, the advent of sequencing, rendering microbial genomes readily accessible, has been of utmost importance for vaccinology and has allowed for highly rational identification of vaccine antigens. Many novel vaccines currently in development are thus based on proteins or peptides predicted by computer databases or by screening antigen libraries^1^,^2^. Although these advances in the post-genomic era have enabled the design of highly pure, safe and simple vaccines, other challenges have emerged in parallel, including the inherent lack of immunostimulatory properties of proteins and peptides. Vaccine adjuvants are therefore considered key components in modern vaccinology since they provide the necessary help of enhancing the immune responses. In addition, adjuvants have many other favorable features including the ability to overcome immune senescence in elderly^3^, prolonging memory of the vaccines^4^, broadening the antibody repertoire^5^ and providing means for dose-sparing^6^. Since the adjuvants currently licensed for human-use almost exclusively induce antibody responses, there is a need for continued adjuvant research and development with the aim of expanding the repertoire of human adjuvants with the ability to induce cell-mediated immunity (CMI).

Early work in adjuvant discovery has been highly empirical and little in depth mechanistic insights is available for the adjuvants currently used in human vaccines. The scarce mechanistic insight is illustrated by the fact that although aluminum salt (commonly referred to as Alum) adjuvants have been used in a range of different vaccines in humans since the 1920s, their mechanism of action remains incompletely understood^7^. The growing insights into innate immunology and the development of a range of novel technical tools have provided a unique opportunity to take adjuvant research and development to a new level, avoiding previous empirical trial-and-error approaches. The recent clinical testing of several novel exploratory adjuvants with the ability to generate CMI responses^8^,^9^ clearly indicates that it is plausible to expand the range of adjuvants available for human use.

In addition to improving the vaccine technology, there is also an urgent need for a more rational preclinical development of adjuvants and better tools for predicting the protective potential of an adjuvant for a given antigen and/or disease target. However, many studies aimed at investigating the potential of novel adjuvants, are carried out with a range of different protocols, different antigens and in many cases with a lack of appropriate comparators e.g. Alum. Studies are also often carried out with non-clinically relevant model antigens, e.g. ovalbumin (OVA), or without animal challenge experiments, which could otherwise provide information on the efficacy. Furthermore, the problems with gaining access to adjuvants currently in clinical development make it difficult to perform comparisons with adjuvants for which we already know the immunological profile in humans. All of these parameters render it impossible to obtain an overview of the potency of different adjuvants and appreciate whether it is worthwhile to pursue further clinical development of a novel adjuvant, hence leaving the adjuvant field fragmented and dispersed^10^. This could potentially undermine valuable information about candidate adjuvants that could otherwise lead to identification of novel viable adjuvants for clinical development. Furthermore, this may lead to overlooking adjuvants with very distinct immunological profiles, which are markedly different from those already filling the clinical pipeline.

Herein, we performed a detailed comparison of five different clinical adjuvants in three different disease models (tuberculosis (TB), chlamydia, and influenza) using a standardized protocol that allowed comparison of results across different antigens and adjuvants. The main objectives of this study were to dissect and compare the immunological profile of these adjuvants and to test the efficacy in very different disease models with different requirements for protection; TB, which primarily requires a Th1 profile^11^, chlamydia for which an antibody response with a mixed Th1/Th17 profile is critical^12^, and influenza where measurement of antibody responses are accepted as a correlate of protection^13^. The proprietary adjuvants included in this study were obtained from partners involved in the EU-funded large collaborative project Advanced Immunization Technologies (ADITEC), and have the common denominator of already being advanced to clinical development (IC31®, CAF01, GLA-SE) or included in registered human vaccines (MF59® and Alum). This provided the possibility of comparing the mouse data reported herein to human data obtained from the clinical studies. The five adjuvants gave rise to very distinct immunological signatures irrespective of the target antigen tested, suggesting that the adjuvants when used together with antigens have potential in induction of protection against highly different diseases. These results on categorization of different clinical-grade adjuvants with proven excellent safety based on their immune profiles and their ability to mount protective immunity, lays the foundation for development of next-generation vaccines for human use.

## Materials and Methods

### Animals

Female CB6F1 (TB and influenza) and B6C3F1 (chlamydia), 6–8 weeks old, were ordered from Harlan Laboratories (The Netherlands, TB and chlamydia) or Charles River (Italy, influenza). TB- and chlamydia-infected mice were housed in animal facilities at Statens Serum Institut, Denmark, while influenza experiments were performed at Novartis Vaccines and Diagnostics s.r.l., Italy. Mice were provided standard food and water *ad libitum*. TB-infected animals were housed in a biosafety level 3 facility in cages contained within laminar flow safety enclosures (Scantainer, Scanbur).

### Ethics Statement

The use of mice was conducted in accordance with the regulations set forward by the respective national animal protection committees and in accordance with European Community Directive 86/609 and the U.S. Association for Laboratory Animal Care recommendations for the care and use of laboratory animals. All the techniques/procedures have been refined to provide for maximum comfort/minimal stress to the animals. Experiments performed at Statens Serum Institut have been approved by the governmental Animal Experiments Inspectorate under licenses 2014-15-2934-01065 (TB) and 2013-15-2934-00978 (chlamydia), while experiments performed at Novartis Vaccines and Diagnostics s.r.l. have been approved by the Ministry of Health under license AEC201111.

### Antigens

#### H56

H56 (Ag85B-ESAT6-Rv2660c) was recombinantly produced in *E. coli* K12 containing the H56 expression vector under IPTG induction, after which the recombinant H56 was harvested as inclusion bodies from disrupted host cells by centrifugation. The inclusion bodies were dissolved in 8 M Urea, 10 mM cysteine, pH 6 and filtered through 0.22 μm filters. The recombinant protein was purified using a two-stage ion exchange scheme (Sartobind® Q SingleSep and S SingleSep), at pH 4.9 and 6.0 respectively) after which the buffer was switched to 20 mM glycine, pH 8.8 using a tangential flow filtration system (Merck Millipore). Protein purity was assessed by SDS-PAGE followed by coomassie staining and western blot with anti-H56 (Polyclonal, rabbit serum) and anti-*E. coli* antibodies (DAKO) to detect contaminants. Furthermore, the protein was validated for residual DNA, endotoxins and bioburden in accordance with internal GMP standards.

#### CT681

A synthetic DNA construct containing ompA (CT681 – AA23-AA349, leaving out the signal peptide) was codon-optimized for expression in *E. coli* followed by insertion into the pJexpress 411 vector (DNA2.0), including a N-terminal histidine tag. Following protein expression, the protein was initially purified by metal chelate affinity chromatography as described in Follmann *et al.* 2008^14^ and subsequently purified by IEC using HiTrap Q Sepharose HP (GE Healthcare). Purified rMOMP was refolded by a stepwise removal of buffer containing urea ending up in 20 mM Tris, 150 mM NaCl, 10% glycerol pH 8 which yielded soluble protein. Protein purity was assessed by SDS-PAGE followed by coomassie staining and western blot with anti-penta-His (Qiagen) and anti-*E. coli* antibodies to detect contaminants (DAKO). The Limulus Amebocyte Lysate test was used to determine the amount of endotoxin in the rMOMP preparation and was below 10 EU/mg.

#### Hemagglutinin (HA)

Influenza A/California/2009 (H1N1) subunit vaccine was produced by Novartis Vaccines. The vaccine was derived from embryonated eggs applying production procedures followed for clinical grade influenza vaccines and its major constituent is hemagglutinin (HA) from the indicated influenza strain.

### Adjuvants

CAF01 from Statens Serum Institut (Copenhagen, Denmark), IC31® from Valneva Austria GmbH (previously Intercell AG, Vienna, Austria), GLA-SE from the Infectious Diseases Research Institute (Seattle, WA, United States), MF59® from Novartis Vaccines (Siena, Italy), Aluminium hydroxide (Al(OH)3) (2% Alhydrogel), from Brenntag Biosector (Frederikssund, Denmark) was used in the TB and Chlamydia experiment, while the Aluminium hydroxide suspension used in the Influenza experiment was produced at Novartis Vaccines and Diagnostics (Marburg, Germany).

### Immunization

**Figure 1:**
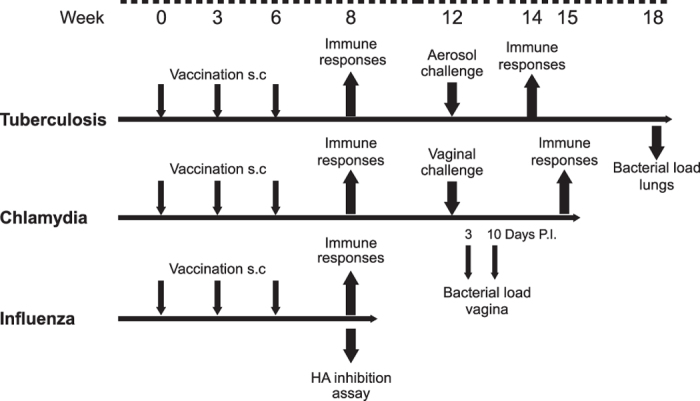
Study design for the animal experiments.

Mice were immunized subcutaneously (s.c.) at the base of the tail three times at three-week intervals. Adjuvants were mixed with 5 μg recombinant antigen or 1 μg of HA by vortexing to a final volume of 200 μl for each injection. CAF01 (dose 250 μg/50 μg (DDA/TDB)/200 μl)^15^, IC31® (dose 100 nmol/4 nmol (KLK/ODN1a)/200 μl)^16^, GLA-SE (dose 5 μg GLA and 2% v/v squalene in 200 μl)^17^, Alhydrogel 2.0% (dose 500 μg aluminum content /200 μl)^18^, MF59® (dose of 100 μl 4.3% w/v squalene, 0.5% w/v Tween 80, 0.5% w/v Span 85 mixed 1:1 with PBS)^19^. Control mice received three injections of 5 μg recombinant antigen or 1 μg HA in 200 μL PBS, respectively, while negative controls received three injections of sterile PBS (pH 7.4). The study designs for the three disease models are shown in Fig. 1.

### ELISA for antigen-specific serum antibodies

Maxisorb Plates (Nunc) were coated with 0.05 μg/well H56 or MOMP in PBS overnight at 4 °C. Individual mouse sera from at least four mice per group were analyzed in duplicate, ten- (IgG1) or fivefold (IgG2a) dilution series, at least 8 times in PBS with 2% BSA, starting with a 20-fold dilution. HRP-conjugated secondary antibodies (rabbit anti-mouse IgG1 and IgG2a; Zymed) was diluted 1:2000 in PBS with 1% BSA. After 1 h of incubation, antigen-specific antibodies were detected using TMB substrate as described by the manufacturer (Kem-En-Tec Diagnostics). The absorbance values were plotted as a function of the reciprocal dilution of serum samples. Reciprocal plasma dilutions corresponding to a cut-off of 0.2 OD450 were computed using Excel (v.15.0.4693.1000). HA specific serum antibodies were determined by a two-step fully automated rapid ELISA (Hamilton Starlet System, Switzerland) with individual sera to titrate total HA specific IgG, IgG1 and IgG2 as described previously^20^.

### Lymphocyte cultures, IL-17 ELISA and Flow cytometry for MOMP and H56

Peripheral blood mononuclear cells (PMBC) and splenocytes were isolated as described previously^21^,^22^. Cultures were adjusted to 2 × 10^5^ cells/well (MSD/ELISA) or 1–2 × 10^6^ cells/well in a total volume of 200 μl/well (IC-FACS) and stimulated with antigens at a final concentration of 2 μg/ml, whereas Con A was used at a concentration of 1 μg/ml as a positive control for cell viability. Culture supernatants were harvested after 72 h of incubation for the investigation of Multiplex cytokine assay and IL-17 ELISA (as previously described^23^). Splenocytes were stimulated at 37 °C in the presence of recombinant antigen (2 μg/ml) for 1 hour, and subsequently incubated for 5 hours after adding 10 μg/ml BFA (Sigma-Aldrich) and stained as previously described^22^. Responses were analyzed using a FACSCanto flow cytometer (BD Biosciences) and FlowJo v.10.0.7 (Tree Star Inc.).

### Intra-cellular flow cytometry staining for splenocytes stimulated *in vitro* with HA antigen

Spleens were harvested and filtered through a 70 μm nylon mesh (BD Biosciences) and stimulated with 1 μg/ml HA O/N and subsequently incubated for 4 hours with BFA (2.5 μg/ml) as described previously^24^. Briefly, cells were stained with combinations of the following antibodies: CD4-V500, CD44-V450, CD3-PerCP-Cy5.5, CD8-Texas Red®, IL-4 and IL-13 Alexa Fluor® 488, IL-2 APC, TNF-Alexa Fluor® 700, IFN-γ PE, IL-17 PE Cy7 and LIVE/DEAD® Fixable Aqua. Flow cytometry was performed on FACS LSRII instruments using DIVA software (BD Biosciences) and data were then analyzed using Flowjo software (Tree Star Inc.).

### Multiplex cytokine assay

The proinflammatory panel 1 (Mouse) 7-plex cytokine assay (IFN-γ, IL-2, IL-4, IL-5, IL-6, IL-10, TNF-α) or the Th1/Th2 panel (mouse) 9-plex cytokine assay (IFN-γ, IL-1b, IL-2, IL-4, IL-5, IL-10, TNF-α, IL-12) or the mouse IL-17 assay (all from Meso Scale Discovery) were performed according to the manufacturer’s instructions. The plates were read on the Sector Imager 2400 system or 6000 system (Meso Scale Discovery) and calculation of cytokine concentrations in unknown samples was determined by 4-parameter logistic non-linear regression analysis of the standard curve.

### Mycobacterial challenge

Six weeks after the third immunization animals were infected with *Mycobacterium tuberculosis* Erdman (ATCC) by the aerosol route as described previously^15^. Six weeks after infection, mice were sacrificed and organs homogenized in PBS for bacterial enumeration as described previously in Ref. 15.

### Chlamydia challenge

Six weeks after the final vaccination the mice were challenged intra-vaginally with 4 × 10^5^ inclusion forming units (IFU) *Chlamydia trachomatis* Serovar D (UW-3/Cx, ATCC VR-885) purchased from the American Type Culture Collection (ATCC) essentially as described previously^15^. The mice oestrus cycles were synchronized ten and 3 days before challenge by subcutaneous injection of 2.5 mg Medroxyprogesteronacetat (Depro-Provera; Pfizer). Vaginal swabs were obtained at day 3 and 10 after infection and analyzed as previously described^18^. Inclusions were enumerated by visual inspection using a fluorescence microscopy.

### Influenza HA inhibition assay

Serum samples were pre-treated with neuraminidase (Biogenetics) overnight at 37 °C, and HI was tested on individual sera. Briefly, 25 μL of two-fold serially diluted samples were incubated with 25 μL of strain-specific influenza antigen (whole virus inactivated, containing four hemagglutinating units) for 60 min at room temperature. A 0.5% (v/v) suspension of adult turkey red blood cells was added and incubated for 60 min. Outcomes were determined by visual inspection: a red dot indicated a positive reaction (inhibition), and a diffuse patch indicated a negative reaction (hemagglutination). All sera were tested in duplicates. The titer was defined as the reciprocal of the serum dilution at which the last complete agglutination inhibition occurred.

### Statistical analysis

Prism 6 software (GraphPad v6.05) was used for all statistical analyses. TB CFU, Chlamydia IFU, IgG and HA inhibition titers were log-transformed before analysis. TB CFU were analyzed using one way ANOVA with Dunnett’s multiple comparisons test. Chlamydia IFU, IgG and HA inhibition titers were analyzed using Kruskal-Wallis test followed by Dunn’s post-test. Statistically significant differences are marked by asterisks in figures and explained in the figure legends.

## Results

### A panel of diverse adjuvants for comparative testing

As part of the EU-funded large collaborative project Advanced Immunization Technologies (ADITEC), several partners contributed with their proprietary adjuvants to create a panel of highly diverse adjuvants already in clinic (Table 1). The panel included squalene-based oil-in-water emulsions (MF59® and GLA-SE – the latter combined with a synthetic TLR4 agonist), cationic peptide KLK combined with the TLR9-stimulatory oligodeoxynucleotide ODN1a (IC31®), cationic liposomes formed of dimethyldioctadecylammonium (DDA) stabilized with synthetic mycobacterial cord factor (trehalosedibehenate) signaling through the C-type lectin receptor Mincle (CAF01), and conventional Alum delivered in the form of a 500 μg aluminum-content dose of diluted 2.0% Alhydrogel. The adjuvants were combined with antigens from three pathogens; H56 is an engineered fusion of three *Mycobacterium tuberculosis* (*M.tb.*) antigens, Ag85B, ESAT-6 and Rv2660c, MOMP is the recombinantly produced major outer membrane protein of *Chlamydia trachomatis* (*C.t.*), and HA is the purified egg-derived hemagglutinin (HA) from A/California/7/2009 (H1N1) influenza strain.

### Comparative evaluation of the humoral immune response induced by the different adjuvants

**Figure 2:**
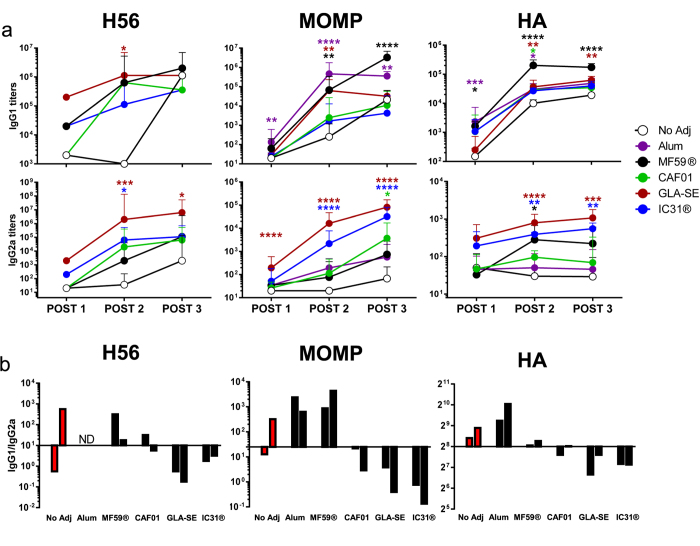
Vaccine-specific humoral immune-responses after vaccination. Groups of mice were immunized 3 times s.c. with H56, MOMP or HA formulated in Alum, MF59® CAF01, GLA-SE or IC31® with 3 week intervals (H56 with Alum was not tested in this assay). Two weeks after each vaccination serum samples from individual mice (n = 4 CB6F1 in TB, n = 12 B6C3F1 in chlamydia, n = 8 CB6F1 in influenza) were analyzed for antigen-specific IgG1 and IgG2a antibodies. Sera from vaccinated animals were analyzed in serial dilutions. H56 and MOMP IgG1/2a titers were determined at a cutoff of 0.2 OD450, while HA-specific IgG1/2a titers were determined from an internal standard. (**a**) IgG1 and IgG2a titers two weeks after 1st, 2nd and 3rd vaccination (Post 1, 2 and 3). Each point represents the geometric mean +95% CI of 4, 12 and 8 mice, for H56, MOMP and HA, respectively. Adjuvanted groups were compared to the no adjuvant group at each time-point using the Kruskal-Wallis test followed by Dunn’s post-test. *P < 0.05; **P < 0.01; ***P < 0.001; ****P < 0.0001. (**b**) The ratio of IgG1/IgG2a titers after the second and third vaccination are shown. Bars represent the geometric means of 4, 12 and 8 mice, for H56, MOMP and HA, respectively. The bars are plotted from the median ratios within each antigen group (H56 10, MOMP 25, HA 256). Post 1 ratios have been omitted due to low titer responses resulting in large variations in ratios. The H56 and MOMP results are representatives of two independent experiments with similar results.

We first compared the ability of the five different adjuvants to induce humoral immune responses using the three model antigens. Groups of mice were vaccinated three times with H56, MOMP, or HA in combination with either CAF01, IC31®, GLA-SE, MF59® or Alum (H56 with Alum was not tested in this assay). Three vaccinations were chosen as previous optimization studies have shown this immunization regimen to induce stronger immune responses compared to two vaccinations using CAF01 (unpublished data) and GLA-SE^25^. Adding a fourth dose did not improve responses (unpublished data). Two weeks after each vaccination, the mice were partially bled and the vaccine-specific IgG1 and IgG2a antibody responses were measured in the serum using ELISA (Fig. 2). The kinetics of specific antibody induction after one, two or three vaccinations showed that GLA-SE in general induced the highest and earliest IgG2a antibody response, with a clear and detectable response after only a single vaccination (Fig. 2a). After the second vaccination, MF59® induced the highest IgG1 antibody response and was clearly superior to the other adjuvants in terms of generating HA-specific IgG1 titers in mice. Compared to vaccination with antigen H56 and MOMP alone (No Adj), there was a clear effect of adding adjuvants on the induction of IgG2a titers in particular after the second and third vaccination. In contrast, the benefit of adding adjuvants on the IgG1 titers was less evident and three vaccinations with recombinant H56 or MOMP alone showed comparable antibody titers as the adjuvanted groups, with the exception of MOMP combined with MF59® or Alum (P > 0.0001 and P > 0.01 compared to No Adj). HA titers followed a slightly different pattern with a more clear benefit of adding adjuvants on IgG1 titers, while the second and third vaccinations, even with adjuvants, had very limited boosting effect on the IgG2a antibody responses after the initial priming. This is in line with previously published data where adjuvanted influenza vaccines are prone towards IgG1 induction while inducing little IgG2a^26^. Common for all three antigens, after the second vaccination and particularly the third vaccination, an IgG1 bias was observed with the Th2-biased adjuvants Alum and MF59® (Fig. 2b), while the Th1-biased adjuvants GLA-SE and IC31® skewed the balance towards IgG2a. CAF01 had a slight bias towards IgG2a induction but with an overall more balanced IgG1/IgG2a ratio. When administered unadjuvanted, all three antigens induced IgG1 biased responses, which is in agreement with previously published data^27^,^28^,^29^.

### Comparative evaluation of the cellular immune responses induced by the different adjuvants

**Figure 3:**
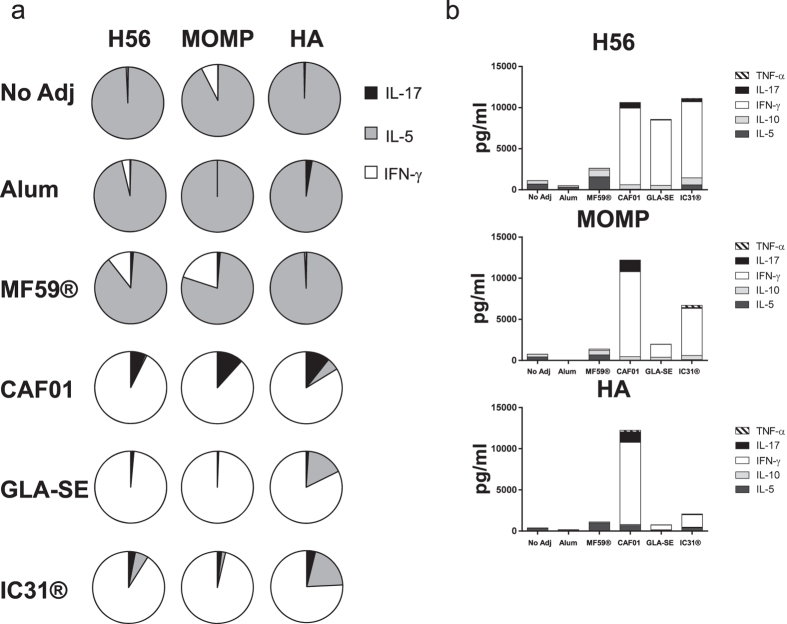
H56, MOMP and HA specific cellular immune response induced by vaccination with different adjuvants. Groups of mice (n = 4 CB6F1 in TB and influenza, B6C3F1 in chlamydia) were vaccinated three times with H56, MOMP or HA formulated in Alum, MF59® CAF01, GLA-SE or IC31®. Two weeks after the third vaccination splenocytes from individual mice were isolated and stimulated with H56, MOMP or HA for 72 hours. The level of cytokines released to the culture supernatants were measured. (**a**) Pie-charts showing the relative contribution of antigen-specific IL-17, IL-5 and IFN-γ. Means of four mice, measured in triplicates and the background from samples stimulated with medium without antigen have been subtracted. (**b**) The levels of IFN-γ, TNF-α, IL-10, IL-5 and IL-17 released to the supernatant are shown as stacked bars, representing the means of four individual mice measured in triplicates and background from samples stimulated with medium without antigen have been subtracted. The H56 and MOMP results are representatives of two independent experiments.

To evaluate CMI responses, splenocytes were isolated two weeks after the third vaccination and re-stimulated with the homologous recombinant protein. The levels of secreted cytokines released to the supernatant after 72 hours of *in vitro* antigen stimulation were measured using MSD technology. Depicting the overall profiles as the relative cytokine contribution demonstrated a breakdown into different classes of adjuvants (Fig. 3a). Thus, Alum and MF59® exhibited a Th2 bias whereas GLA-SE and IC31® induced a clear Th1-biased response with a strong IFN-γ component and negligible levels of IL-5. CAF01 induced a mixed Th1/Th17 phenotype and was the only adjuvant with a clear IL-17 response for all three antigens. Considering the absolute amount of cytokine secretion showed a more diverse pattern and magnitude depending on the type of antigen used (Fig. 3b). Thus, CAF01 was the only adjuvant consistently giving rise to high levels of cytokine secretion, primarily IFN-γ, with all three antigens whereas IC31® gave highest response in combination with H56, intermediate levels with MOMP and low amounts in combination with HA. GLA-SE had a prominent adjuvant effect for H56 whereas it showed a modest effect in adjuvanting HA and MOMP.

The phenotype and cytokine subsets were further assessed by flow cytometry using the frequency of IFN-γ, IL-17 and IL-4/IL-13 for HA (Fig. S1) and the IFN-γ, IL-2, TNF-α distribution for H56 and MOMP (Fig. S2). Common for all adjuvants was the induction of CD4 T cell responses whereas no CD8 T cell responses were observed (results not shown). All groups receiving HA showed a measurable IL-4/IL-13 population, suggesting a Th2-bias response to this antigen in CB6F1 mice. This Th2 response was increased in combination with all adjuvants, albeit groups receiving HA in combination with the Th1 adjuvants, GLA-SE, IC31®, and CAF01 also had a population of IFN-γ producing CD4 T cells. CAF01 induced the overall highest recall response upon restimulation with HA and an additional IL-17 producing component. Overall, the phenotypes determined by flow cytometry were coherent with those determined by MSD. In the H56/MOMP analysis, IC31® induced the most vigorous response in terms of frequency of cytokine-producing CD4 T cells in particular when combined with H56. The response was dominated by triple-positive IFN-γ/IL-2/TNF-α T cells constituting ~50% of the cells, but also a considerable population of IL-2/TNF-α double producing cells usually considered to be the key trait of central memory T cells^22^. GLA-SE and CAF01 induced comparable T cell profiles with triple-positive, TNF-α/IL-2 double positive and TNF-α single producers. Furthermore, IFN-γ/TNF-α double producers were observed with CAF01, GLA-SE and IC31®, while IC31® was the only adjuvant that induced a small but noticeable population of IFN-γ single producers. IFN-γ/TNF-α and IFN-γ single producers are generally considered as more differentiated/effector subpopulations^30^. MF59® induced a strong Th2 bias response, based on the IL-5 secretion (see Fig. 3a,b), as well as distinct populations of TNF-α single producing CD4 T cells and IL-2/TNF-α co-producers.

### *In vitro* evaluation of protective efficacy against influenza

In order to assess the adjuvants ability to induce protective immunity against influenza, the hemagglutination inhibition (HAI) titers were measured two weeks after each vaccination (Table 2). The HAI titer is a surrogate marker of influenza-specific neutralizing antibodies widely used for *in vitro* correlate of protection^13^. MF59® adjuvanted HA induced the highest levels of HAI titers, significantly higher than that of HA alone after the second vaccination (p < 0.0001, Kruskal-Wallis followed by Dunn’s post-test compared to no adjuvant). GLA-SE also elicited a significantly elevated HAI titer after the second vaccination (p < 0.05) while IC31®, CAF01 and Alum did not induce significantly higher HAI titers compared to that of antigen alone.

### Protective efficacy in an aerosol TB challenge model

**Figure 4:**
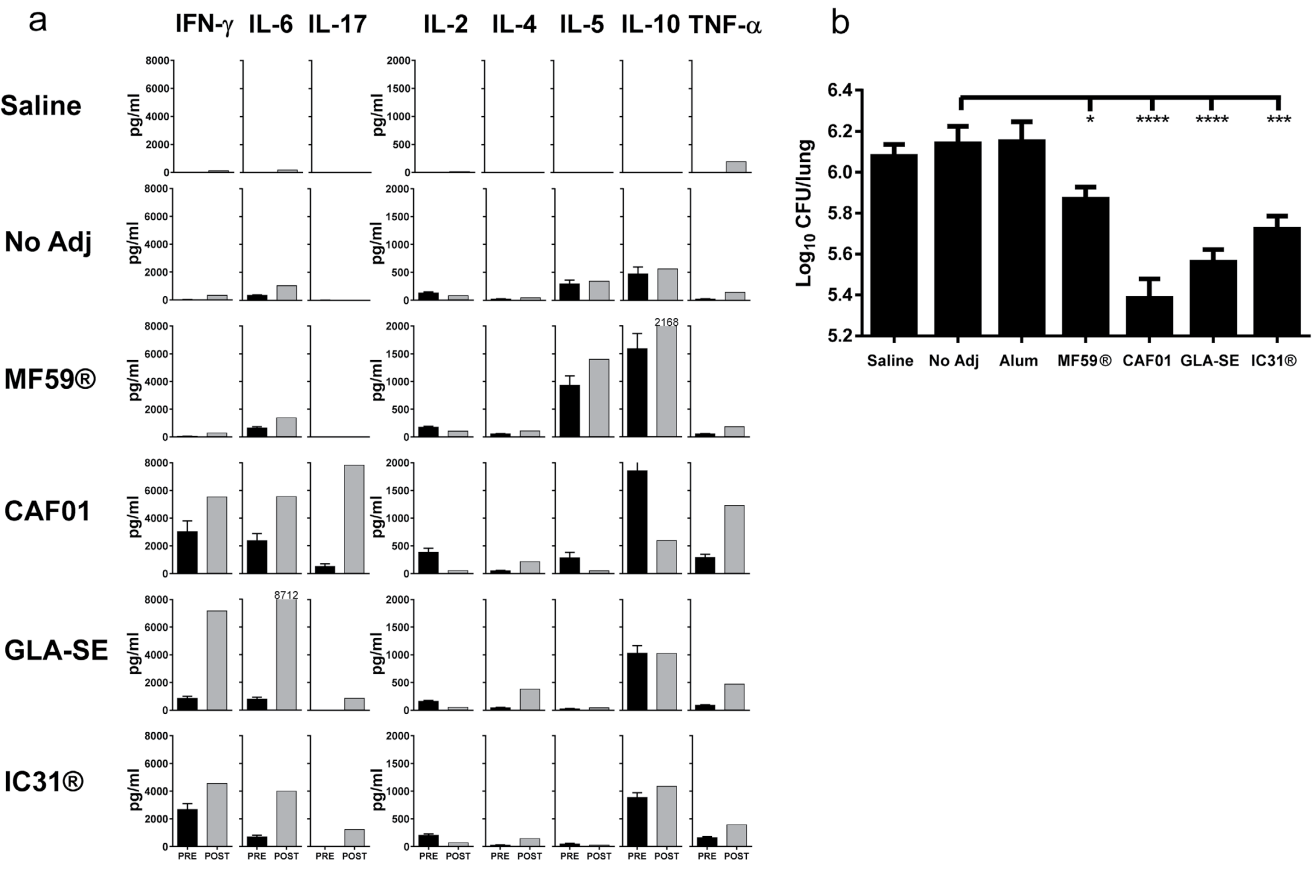
H56 specific immune responses and protective efficacy against challenge with *Mycobacterium tuberculosis*. Groups of mice (CB6F1) were immunized three times s.c. with 5 μg of H56 formulated in Alum, MF59® CAF01, GLA-SE or IC31® with 3 week intervals. Six weeks after the third vaccination, mice received an aerosol challenge with *M.tb.* Erdman (~50 CFU/mouse). Two weeks after challenge, PBMC’s were isolated from a pool of 12 individual mice and stimulated with recombinant H56 (2 μg/ml) for 72 hours. Culture supernatants were analyzed for released cytokines (TNF-α, IFN-γ, IL-2, IL-4, IL-5, IL-6, IL-10, IL-17). (**a**) Grey bars indicate the levels of cytokines released from PBMCs after infection (Post) and the black bars represent the levels of cytokines measured in splenocytes prior to infection (Pre). Black bars represent mean + SEM of four mice measured in triplicates (Pre), while grey bars are means of triplicates measured of PBMC’s pooled from 12 mice within groups (Post). (**b**) After six weeks of infection, mice were euthanized and the bacterial loads in the lungs (CFUs) of individual mice was assessed. Each bar represents the mean + SEM Log_10_ CFU levels and represents 14–16 mice (Alum n = 8). The results are pooled from two experiments with same overall results. Adjuvanted groups were compared to the “no adjuvant” group using one way ANOVA with Dunnett’s multiple comparisons test. *P < 0.05; ***P < 0.001; ****P < 0.0001.

The potential of different adjuvants in induction of protective immunity to M.tb. infection was then studied. Six weeks after the third vaccination, H56 vaccinated mice were challenged with *M.tb*. Erdman through the aerosol route. Two weeks after challenge, PBMCs were isolated and stimulated with H56 for 72 hours and analyzed for vaccine-specific secretion of IFN-γ, IL-10, IL-2, IL-4, IL-5, IL-6, TNF-α and IL-17 (Fig. 4a). The post-infection H56 responses measured in the PBMC’s (post) were compared to the pre-infection responses in the splenocytes (pre) with the differences in target organ kept in mind. While infection alone induces limited responses at this early time point after challenge, all groups previously primed with an adjuvanted vaccine were able to mount significant responses. H56 in combination with CAF01, IC31® and GLA-SE all primed IFN-γ, IL-6 and TNF-α responses prior to infection and all of these cytokine responses remained two weeks after challenge. The strongest amplification of IFN-γ and IL-6 responses was observed in the GLA-SE vaccinated group. MF59® maintained the same profile with no IFN-γ secretion after infection. CAF01 was the only group where an IL-17 response was observed after immunization and this response was strongly elevated after infection. In contrast, the IL-17 response was induced only after infection in the IC31® and GLA-SE groups. H56-specific IL-10 was seen in all groups independent of their Th-profile and was not augmented by the infection.

Six weeks after infection, the mice were sacrificed and the numbers of viable colony-forming units (CFU) in the lungs were enumerated in order to assess protective efficacies. (Fig. 4b). The highest levels of protection were seen upon vaccination with H56 in CAF01/GLA-SE (p < 0.0001) and IC31® (p < 0.001), whereas MF59® gave rise to a relatively modest, but significant, reduction of 0.27 log_10_ CFU (p < 0.05). As with the related fusion protein H1 (Ag85B-ESAT-6), H56 combined with Alum did not mount protective immunity to TB challenge with comparable levels of bacterial numbers as the H56 alone group^15^. There was no significant difference between the PBS control group and H56 antigen alone (No adj).

### Protective efficacy in a murine chlamydia challenge model

**Figure 5:**
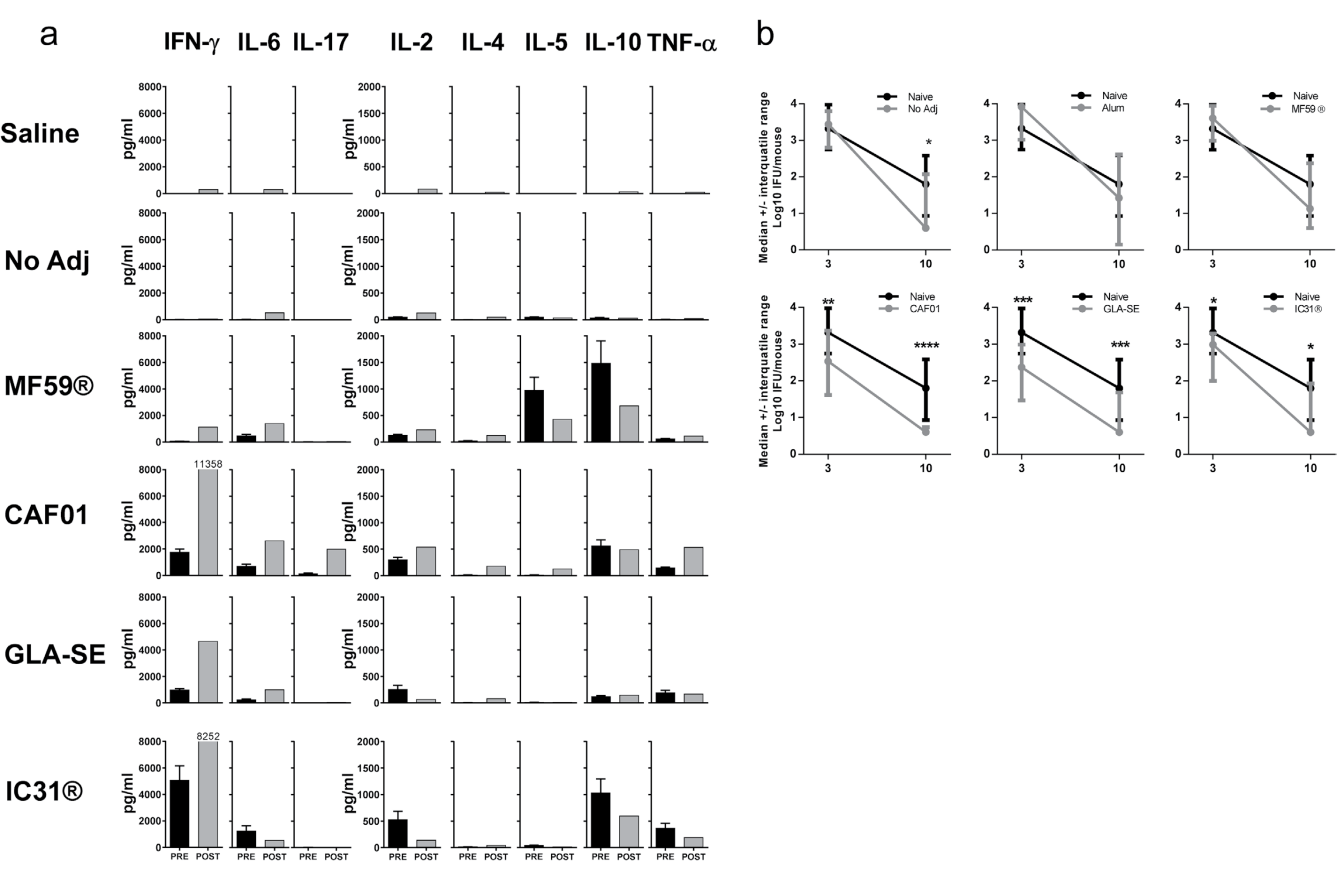
MOMP specific immune responses and protective efficacy against challenge with *Clamydia trachomatis*. Groups of mice (B6C3F1) were immunized three times s.c. with 5 μg of MOMP formulated in Alum, MF59® CAF01, GLA-SE or IC31® with 3 week intervals. Six weeks after the third vaccination, mice received a vaginal challenge with *C. trachomatis*. Three weeks after challenge, PBMC’s were isolated from individual mice (n = 12), pooled within groups and stimulated with recombinant MOMP for 72 hours. Culture supernatants were analyzed for released cytokines (TNF-α, IFN-γ, IL-2, IL-4, IL-5, IL-6, IL-10, IL-17). (**a**) The levels of cytokines released from PBMCs after infection (post) were compared to the levels of cytokines prior to infection, measured in splenocytes (pre). Black bars represent mean + SEM of four mice measured in triplicates (Pre), while grey bars are means of triplicates measured of PBMC’s pooled from 12 mice within groups (Post). (**b**) On day 3 and 10 after infection, mice were vaginally swabbed and the vaginal bacterial load (IFUs) of 28 individual mice (Naïve n = 56) was assessed. Each point represents the median Log_10_ ± interquatile range. Adjuvanted groups were compared to the naïve controls at each time-point using the Kruskal-Wallis test followed by Dunn’s post-test. *P < 0.05; ***P < 0.001; ****P < 0.0001.

Next, we sought to evaluate the efficacy of different vaccine formulations in induction of protective immunity to a vaginal *C.t.* challenge. Groups of mice, vaccinated with MOMP singly or in combination with different adjuvants, were challenged vaginally with *C.t.* six weeks after the last vaccination. Three weeks after challenge, PBMCs were stimulated with recombinant MOMP for 72 hours for analysis of IFN-γ, IL-10, IL-2, IL-4, IL-5, IL-6, TNF-α and IL-17 in the supernatant. The post-infection PBMC responses (post) as well as the pre-infection splenocyte responses were examined (pre) (Fig. 5a). Like for H56, the cytokines IFN-γ, IL-6 and TNF-α were among the dominating cytokines in the CAF01, GLA-SE, and IC31® groups but only IFN-γ seemed to be selectively increased upon chlamydia infection. The CAF01-induced IL-17 response was shown to be clearly boosted by the infection, although not to the same extent as that observed with the TB infection and further, no IL-17 responses are seen in any other groups.

Vaginal swabs were collected at day 3 and 10 post challenge and protection was assessed by isolating *C.t.* from vaginal swabs and comparing the number of IFUs recovered at the indicated time points (Fig. 5b). CAF01, IC31® and GLA-SE were able to significantly reduce the bacterial shedding at day 3 and 10 post challenge, whereas MOMP adjuvanted with MF59® or Alum was not able to induce significant protection at any time-point. The un-adjuvanted MOMP vaccinated group did not show any significant reduction in their bacterial loads on day 3 after challenge, albeit a significant reduction was seen at day 10 post challenge.

## Discussion

Herein, we report for the first time a comprehensive head-to-head comparison of immunogenicity and protective efficacy of five different clinically tested/practiced adjuvants; Alum, MF59®, CAF01, GLA-SE and IC31®. The adjuvant panel is highly diverse, but with the common denominator of having been tested in human subjects offering a unique opportunity to compare the mouse data from the current study with already available human data. Alum was the first adjuvant to be used in human vaccines and since the 1920s, billions of doses have been administered primarily with the purpose of adsorbing antigen and inducing antibodies. From numerous human trials, it is known that Alum is a poor inducer of cellular immune responses. In line with the human data and as reported previously in several animal studies, the single characteristic of Alum in our study was its ability to enhance antibody titers. MF59® has been used in licensed vaccines in Europe since 1997 and primarily induces antibodies and an increased CD4 T cell response with an unbiased Th-profile in humans^31^,^32^,^33^. Overall, this is in agreement with our findings in mice showing strong antibody responses but also induction of a CD4 response with a Th2 profile along with CD4 T cells co-producing TNF-α/IL-2. GLA-SE, CAF01, and IC31® are in the early stages of clinical development but it is clear from the emerging data that they are able to induce strong and long-lived CMI responses in humans and for CAF01 and IC31® phenotypic profiles with a remarkable resemblance between mouse and man.

MF59® was originally developed as a delivery system for immunomodulators but pre-clinical studies suggested that this emulsion had potential on its own with influenza antigens^34^. MF59® was the most effective adjuvant in an influenza vaccine assessed as increased IgG1 and protective HI titers and indeed numerous clinical trials in humans show the same overall profile and also a clear efficacy of MF59® in influenza vaccines^35^ (reviewed by Reed *et al.* 2013^36^). However, in humans with no pre-existing immune response e.g. against H5N1, two vaccinations seem to be required in order to enhance IgG titers (Galli *et al.* 2009, Fig. 2^37^). Since the discovery of the adjuvant activity of aluminum compounds, there have been several attempts to use different aluminum forms for augmenting the effect of influenza vaccines but with very limited success^38^ (reviewed by Tetsutani *et al.* 2012^39^). In a clinical study directly comparing the effect of Alum and MF59®, there was an 8–9 fold increase in frequency of subjects reaching the predefined endpoint (HAI titers ≥ 40), when vaccinated with equivalent doses of subvirion influenza A/H5N1 in MF59® compared to Alum^40^. The superiority of MF59® over Alum in induction of influenza specific antibody responses was also observed in our study with a 2.5–10 fold increase in HAI for MF59® compared to Alum and in a recent study aimed at investigating the efficacy of emulsion adjuvants in influenza subunit vaccines^24^.

It is well-known that the antibodies raised by current inactivated influenza vaccines are mainly strain-specific antibodies directed against highly variable surface proteins resulting in protection against antigenically matching virus strains. With the threat of novel pandemic influenza variants and the continuous emergence of drifted strains, there has been considerable interest in the development of influenza vaccines with broader coverage. Several clinical trials have shown that MF59® can induce responses of increased breadth compared to un-adjuvanted influenza vaccines, which mainly relates to the antibody repertoire, whereas there is limited information on the ability of MF59® to induce CMI responses to the vaccine (reviewed in O´Hagan *et al.* 2011^41^). Thus, it has been suggested that the potency of emulsion-based adjuvants could be improved by combining them with additional immunomodulators capable of inducing CD8 and/or CD4 T cell responses, with the ability to induce influenza cross-protection^42^,^43^. Accordingly, it has been shown in preclinical studies that GLA-SE containing the TLR4-ligand GLA, and not the stable emulsion (SE) alone, was capable of inducing protection against infection with a heterologous virus strain^44^. As in the studies herein, GLA-SE was found to enhance IFN-γ secretion and induce a shift from IgG1 to IgG2 production. In our study, GLA-SE also elicited significantly elevated HAI titers, which is in line with recent human data where GLA-SE induced HAI titers ≥1:40 in more than 66% of the subjects^45^. In the direct comparison of the HAI titers against the homologous virus strains, MF59® gave rise to stronger responses but it is plausible that changing the experimental set-up e.g. assessing survival upon challenge with a heterologous strain would offer advantages to GLA-SE.

The adjuvants IC31® and CAF01 were developed with the purpose of generating Th1 responses and both induce lower HAI titers in direct comparison to emulsion-based adjuvants. Paralleling the design of GLA-SE, it may be possible to benefit from their Th1-inducing ability e.g. by combining their individual immunomodulators into a stable emulsion formulation. Although the major purpose would be to augment the induction of neutralizing antibodies, it is important to maintain their ability to generate memory T cells that not only recognize conserved viral proteins, and thereby provide cross-protective responses, but also provide long-lived protection, which is of particular relevance for prophylactic vaccines. In this context, CAF01 in combination with trivalent inactivated vaccines (TIV) has previously been shown to promote significant T cell responses in the ferret model, which is considered to be the most accepted model of influenza disease, and importantly provided higher levels of protection compared to TIV alone and even TIV in a squalene emulsion^46^. A detailed characterization of the murine immune response afforded by a trivalent influenza vaccine administered in IC31® showed a very pronounced induction of IFN-γ that was maintained even 200 days after a single vaccination, at least in part, due to the formation of a vaccine depot at the injection site^47^,^48^.

CAF01-based TB vaccines were also capable of inducing long-term protective responses with a high frequency of vaccine-induced CD4 T-cells retrieved more than one year after vaccination in mice^4^. The T-cells had a strong proliferative potential presumably facilitated by the specific physicochemical properties of the delivery vehicle (DDA) that supports a slow-release of antigen from the site of vaccination^49^. The immunogenicity results from human trials have so far been comparable for IC31® and CAF01, both of which were capable of inducing very persisting T cell responses as late as 2½ (IC31®^8^) and 3 years (CAF01^9^) after vaccination. Assessing the phenotype of the vaccine-induced CD4 T-cells at this late-stage post vaccination showed that more than 50% of the cells co-secreted TNF-α and IL-2; a phenotype which has been associated with central memory T cells (T_cm_)^22^. In our studies, all three adjuvants with the ability to induce highly significant protection against TB, induced phenotypic profiles very similar to what was observed previously in humans with a substantial proportion of IL-2/TNF-α co-producers, most pronounced in the CAF01 group where app. 50% of the cells were of this phenotype. Indeed, of the relatively few Th1 cells induced by MF59®, the majority were IL-2/TNF-α co-producers, which were reported to represent the main population induced by MF59® adjuvanted influenza vaccine in children^33^, and this could explain the modest, albeit significant, protection induced by a MF59®-adjuvanted TB vaccine. There is an increasing amount of evidence that T_cm_’s may play an important role in protection against TB. Most recently, the Kaufmann lab demonstrated that the protection afforded by a recombinant BCG primarily resided in the T_cm_ population^50^, but also in humans this phenotype was shown to be associated with responses in individuals capable of controlling infection^51^. Whether this profile directly mediates the superior TB disease control in humans, including vaccine-promoted protection, remains to be investigated.

Whereas there is concordance between murine and human data in regards to effector and memory T cell phenotypes, the specific induction of IL-17 producing CD4 T cells seen upon vaccination with the CAF01 adjuvant in this and several other studies is not directly mirrored in humans. In a recent phase I study, vaccination with the TB fusion-protein H1 in CAF01 was unable to induce high levels of IL-17 following two immunizations in healthy individuals^9^. In this study, we compared the post vaccination splenic responses to post infection PBMC’s, and although cytokine levels from these two different compartments are not directly comparable, some key differences between the adjuvants became evident from the relative comparison between groups. The vaccine-induced IL-17 primed by CAF01 vaccination was strongly boosted upon *M.tb.* infection (Fig. 4a). In groups vaccinated with GLA-SE or IC31®, no apparent IL-17 responses were detected after vaccination in the splenocytes, but upon *M.tb.* infection both exhibited measurable IL-17 responses in the PBMC’s. It is possible that CAF01-based vaccines are also able to induce IL-17 in humans but at a very low and un-detectable level and that the response only becomes apparent after infection. This boosting was not observed with MF59®, H56 alone, or in the saline control group. The immunomodulator in CAF01, TDB, was shown to signal through the C-type lectin receptor Mincle in mice, which in turn elicits a unique Th1/Th17-biased immune response through the Syk-Card9 signaling pathway^52^. Mincle is expressed on a variety of cell types e.g. neutrophils, dendritic cells, and macrophages and a human variant of the Mincle receptor has also been identified^53^. The recent study showing that TDB is capable of binding to human Mincle and activate APCs *in vitro*^54^ indicates that Mincle-activation may also be important for induction of immune response in humans. However, the use of more sensitive techniques and studying more immunologically competent compartments other than the PBMCs might be needed to detect Th17 responses in humans.

It is well-established that CMI responses, and in particular IFN-γ producing CD4 T-cells, play a major role in protection against chlamydia infection^55^,^56^. In agreement with this, vaccination with all three Th1 adjuvants (IC31®, GLA-SE, CAF01) reduced bacterial shedding in the genital tract at both day 3 and 10 (Fig. 5b). Testing different adjuvants, Dr. Robert Brunham’s lab demonstrated a superior effect of an adjuvant-formulation giving rise to a combined IFN-γ/IL-17 response^57^, however in our studies we did not see a superior effect of the IL-17 inducing CAF01 compared to the sole Th1 inducing adjuvant GLA-SE. The Th2 inducing adjuvant MF59® in combination with MOMP had no protective effect, which is in line with previous reports showing reduced migration to the genital mucosa of a chlamydia-specific Th2 clone compared to its Th1 counterpart^58^. It has been shown that antibodies play an important role for providing early clearance of infection using the same chlamydia model as in this study^59^ as well as against reinfection^60^. Somewhat surprising, we could observe that three vaccinations with un-adjuvanted MOMP induced an antibody response comparable to those of adjuvanted MOMP, which may explain the significant protection observed at day 10 in the un-adjuvanted MOMP group.

While it is generally accepted that a combination of humoral and cell mediated immunity is protective against chlamydia and that protection when using HI titers as influenza readout is mediated by a humoral response, the contribution of antibodies to protection against *M.tb.* is still debated. The comparison of the IgG1/2a titers (Fig. 2a) and TB bacterial burdens (Fig. 4b) indicates that protection against TB does not depend on antibody induction. While this fits with the general notion that a CMI Th1 response is crucial for TB protection, it does not elaborate on the possibility of a synergetic effect between cell mediated and humoral immunity. In particular, the induction of IgA titers by vaccination might be relevant as this isotype previously has been associated with protection against TB^61^. Currently, there are no vaccines building on the concept of inducing humoral immune responses in the clinical TB pipeline but this specific topic has generated interest from the funding bodies and it is possible that we will see new types of TB vaccines in the coming years.

It should be noted that the head-to-head evaluation of a wide range of different adjuvants for their ability to enhance immunogenicity of different antigens is a challenging task. In this regard, it is highly satisfying to observe that each individual adjuvant produced similar antigen-specific immunological profiles for three unique vaccine antigens. Thus, the distribution of T-cell phenotypes are the same whether using e.g. a TB antigen or the chlamydia MOMP protein, MF59® induces IL-5 independent of the antigen of choice, and GLA-SE generates the highest IgG2a titers with antigens produced in *E. coli* as well as with an antigen produced by embryonated eggs. Despite this remarkable consistency in adjuvant activity across different pathogen targets, there are, however, some key limitations in the current study that need to be taken into account; In order to perform this head-to-head comparison a standardized protocol was employed across the three models. This included unifying the number of vaccinations, amount of antigen, dose of adjuvant, timing between vaccinations, administration route and resting period before challenge and subsequent determination of protection. Nevertheless, all of these factors would presumably have a different optimum for different combinations of adjuvants and antigens, implying that some of these factors might not have been optimal for the tested adjuvants, hence preventing the adjuvants to exert their full adjuvanticity in the head-to-head comparison. However, due to the consistency in the immunological profiles of the different adjuvants across antigens, which are in line with the previously published human data, we are confident that these results could inform rational selection of suitable adjuvants to mount desired protective immune responses.

To our knowledge, this is the first report of a comprehensive non-clinical comparison of clinically tested/practiced adjuvants from different stakeholders, including academia and the private sector, and it is our hope that results obtained from this study will provide a platform for how adjuvants should be compared and thereby help accelerating adjuvant research and development.

## Additional Information

**How to cite this article**: Knudsen, N. P. H. *et al.* Different human vaccine adjuvants promote distinct antigen-independent immunological signatures tailored to different pathogens. *Sci. Rep.* **6**, 19570; doi: 10.1038/srep19570 (2016).

## Supplementary Material

Supplementary Information

## Figures and Tables

**Table 1:** 

Adjuvant	Category	Immune profile*	Stage of development	References
Alum	Insoluble aluminum salts	T_H_2, antibody	In several licensed products	62,63
MF59^®^	Squalene oil-in-water emulsion	(T_H_1), T_H_2, antibody	Licensed for use in influenza vaccine in EU	64,65,66
CAF01	Cationic liposomes formulated with a Mincle agonist	T_H_1, T_H_17	Phase 1	45,67
GLA-SE	Squalene emulsion combined with a TLR4 agonist	T_H_1, antibody	Phase 2	9,68
IC31^®^	Cationic antimicrobial polypeptides combined with a TLR9 agonist	T_H_1	Phase 2	8,69

*Immune profile reported in the literature, see references.

**Table 2:** 

HA inhibition titers						
Adjuvant	POST 1		POST 2		POST 3	
	GMT	95% CI	GMT	95% CI	GMT	95% CI
PBS	<10	*—*	<10	*—*	<10	*—*
No Adj	62	[19–203]	1050	*[434*–*2543]*	1076	*[548*–*2113]*
Alum	538^*^	*[227*–*1274]*	1810	*[1327*–*2468]*	2915	*[1636*–*5198]*
MF59^®^	320	*[133*–*768]*	19612^****^	*[10087*–*38177]*	8611^****^	*[5890*–*12582]*
CAF01	336	*[117*–*800]*	2690	*[2143*–*3964]*	2436	*[2213*–*2716]*
GLA-SE	269	*[179*–*405]*	5346^*^	*[3242*–*8822]*	5263^*^	*[2078*–*13333]*
IC31^®^	381	*[145*–*1001]*	2792	*[1765*–*4416]*	2560	*[1878*–*3491]*
