# Supplementary material for: Different human vaccine adjuvants promote distinct antigen-independent immunological signatures tailored to different pathogens
Source: Sci Rep. 2016 Jan 21;6:19570. doi: 10.1038/srep19570 (PMC4726129; doi:10.1038/srep19570)

Different human vaccine adjuvants promote distinct antigen-independent immunological signatures tailored to different pathogens

Niels Peter H. Knudsen[a]*, Anja Olsen[a], Cecilia Buonsanti[b], Frank Follmann[a], Yuan Zhang[c], Rhea N. Coler[d], Christopher B. Fox[d], Andreas Meinke[e], Ugo D´Oro[b], Daniele Casini[b], Alessandra Bonci[b], Rolf Billeskov[a], Ennio De Gregorio[b], Rino Rappuoli[b], Ali M. Harandi[c], Peter Andersen[a] and Else Marie Agger[a]*

[a]Department of Infectious Disease Immunology, Statens Serum Institut, Copenhagen, Denmark

[b]Novartis Vaccines and Diagnostics s.r.l (a GSK Company), Siena, Italy

[c]Department of Microbiology and Immunology, University of Gothenburg, Gothenburg, Sweden

[d]Infectious Disease Research Institute, Seattle, WA, USA

[e]Valneva Austria GmbH, Vienna, Austria

*Corresponding Authors

Niels Peter H. Knudsen,	NPK@ssi.dk
Anja Olsen,	AOL@ssi.dk
Cecilia Buonsanti,	cecilia.x.buonsanti@gsk.com
Frank Follmann,	FRF@ssi.dk
Yuan Zhang,	yuan.zhang@gu.se
Rhea N. Coler,	Rhea.Coler@idri.org
Christopher B. Fox,	Chris.Fox@idri.org
Andreas Meinke,	Andreas.MEINKE@valneva.com
Ugo D´Oro,	ugo.x.d'oro@gsk.com
Daniele Casini,	daniele.x.casini@gsk.com
Alessandra Bonci,	alessandra.x.bonci@gsk.com
Rolf Billeskov	ROB@ssi.dk
Ennio De Gregorio,	ennio.de_gregorio@novartis.com
Rino Rappuoli,	rino.r.rappuoli@gsk.com
Ali M. Harandi,	ali.harandi@microbio.gu.se
Peter Andersen,	PA@ssi.dk
Else Marie Agger,	EAG@ssi.dk

Figure 1S. icFACS analysis of vaccine responses measured by cytokine expression in HA specific CD4 T-cells. Mice were vaccinated 3 times s.c. with 1 μg of HA formulated in Alum, MF59® CAF01, GLA-SE or IC31® with 3 week intervals. Splenocytes from four individual mice, isolated two weeks after the third immunization, were stimulated with 1 μg/ml of recombinant HA O/N + 4h in the presence of BFA. Cells were gated as follows: live > singlets > lymphocytes > $CD3^+$ > $CD4^+$ versus $CD8^+$. Cytokine-producing cells (IFN-γ, IL-17 or IL-4/IL-13) within the $CD4^+CD44^{high}$ population were measured. Bar charts show the frequency of each cytokine subset being $CD44^{high}$ out of the total CD4 T cell population. Bars represent the mean + SEM of four individual mice. The background from non-stimulated samples have been subtracted.

Figure 2S. An icFACS analysis of vaccine responses measured by cytokine co-expression in H56 or MOMP-specific CD4 T-cells. Mice were vaccinated 3 times s.c. with 5 μg of H56 or MOMP formulated in Alum, MF59® CAF01, GLA-SE or IC31® with 3 week intervals. Splenocytes from four individual mice, isolated two weeks after the third immunization, were stimulated with 2 μg/ml of recombinant H56 or MOMP for 1h + 5h in the presence of BFA. Cells were gated as follows: singlets > lymphocytes > $CD4^+$ versus $CD8^+$. Cytokine-producing cells (IFN-γ, TNF-α, and IL-2) within the $CD4^+CD44^{high}$ population were divided into seven distinct subpopulations based on Boolean gating and are shown as bar and pie charts. Bar charts show the frequency of each cytokine subset being $CD44^{high}$ out of the total CD4 T cell population. Bars represent the mean + SEM of four individual mice. Pie charts show the relative contribution of each cytokine subpopulation to the Ag-responsive CD4 T cell population. The background from non-stimulated samples have been subtracted. The experiment was repeated with similar results.

Supplementary Figure S1

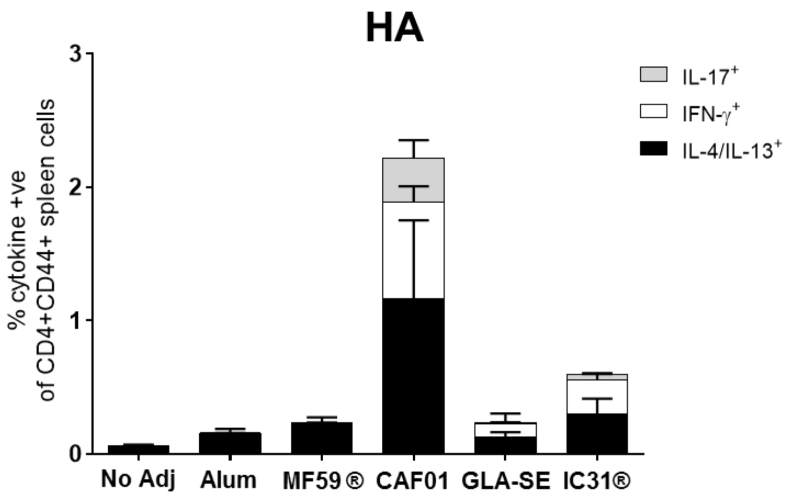

Supplementary Figure S2

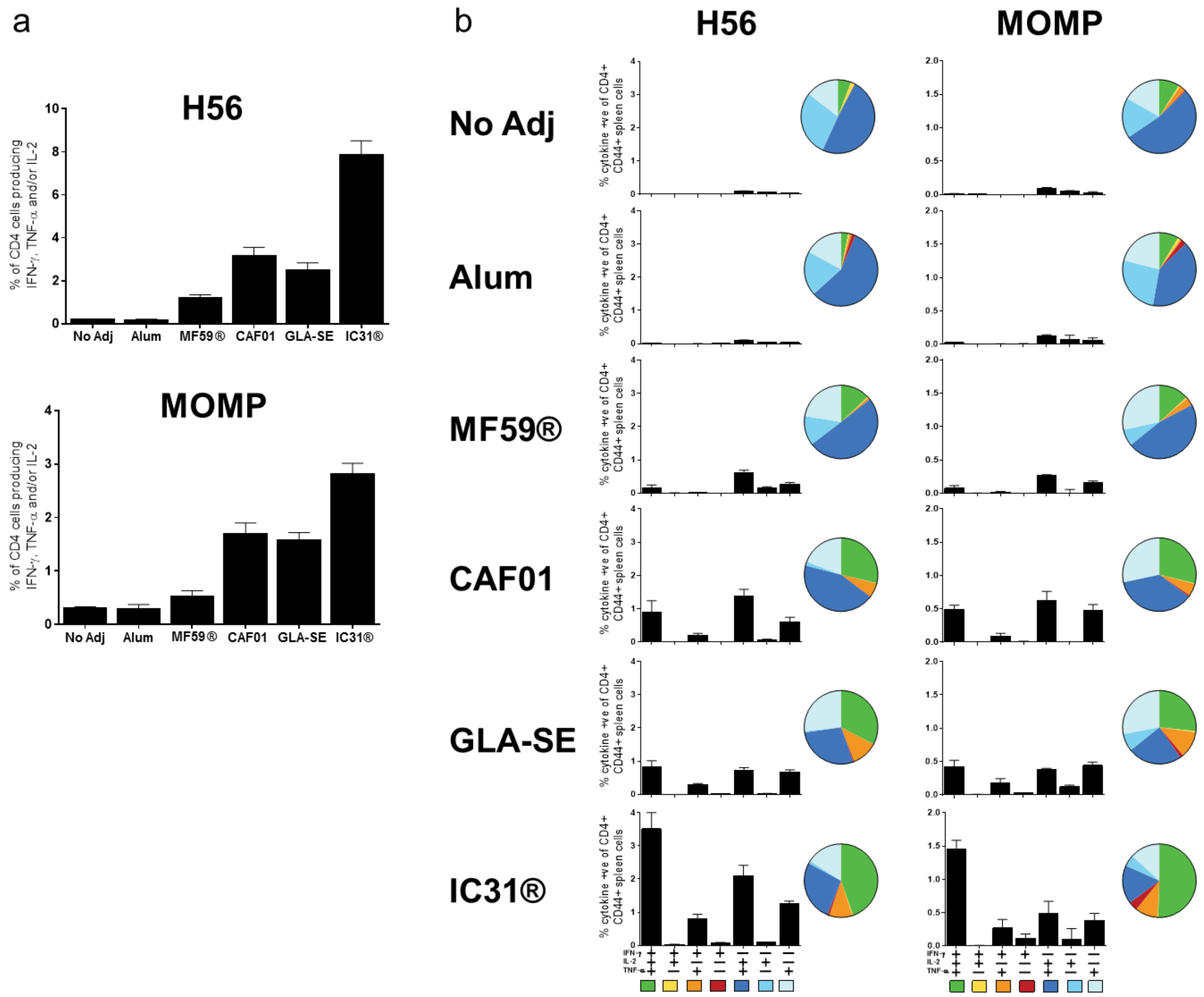

Supplement: Supplementary Information [file srep19570-s1.pdf]
